# Multiscale and Multiphysics Modeling of Anisotropic Cardiac RFCA: Experimental-Based Model Calibration *via* Multi-Point Temperature Measurements

**DOI:** 10.3389/fphys.2022.845896

**Published:** 2022-04-19

**Authors:** Leonardo Molinari, Martina Zaltieri, Carlo Massaroni, Simonetta Filippi, Alessio Gizzi, Emiliano Schena

**Affiliations:** ^1^ Department of Mathematics and Computer Science, Emory University, Atlanta, GA, United States; ^2^ Laboratory of Measurement and Biomedical Instrumentation, Department of Engineering, University of Rome Campus Bio-Medico, Rome, Italy; ^3^ Nonlinear Physics and Mathematical Modeling Lab, Department of Engineering, University of Rome Campus Bio-Medico, Rome, Italy

**Keywords:** radiofrequency ablation, myocardial anisotropy, hyperthermal tissue damage, fiber Bragg grating sensors, finite element analysis

## Abstract

Radiofrequency catheter ablation (RFCA) is the mainstream treatment for drug-refractory cardiac fibrillation. Multiple studies demonstrated that incorrect dosage of radiofrequency energy to the myocardium could lead to uncontrolled tissue damage or treatment failure, with the consequent need for unplanned reoperations. Monitoring tissue temperature during thermal therapy and predicting the extent of lesions may improve treatment efficacy. Cardiac computational modeling represents a viable tool for identifying optimal RFCA settings, though predictability issues still limit a widespread usage of such a technology in clinical scenarios. We aim to fill this gap by assessing the influence of the intrinsic myocardial microstructure on the thermo-electric behavior at the tissue level. By performing multi-point temperature measurements on *ex-vivo* swine cardiac tissue samples, the experimental characterization of myocardial thermal anisotropy allowed us to assemble a fine-tuned thermo-electric material model of the cardiac tissue. We implemented a multiphysics and multiscale computational framework, encompassing thermo-electric anisotropic conduction, phase-lagging for heat transfer, and a three-state dynamical system for cellular death and lesion estimation. Our analysis resulted in a remarkable agreement between *ex-vivo* measurements and numerical results. Accordingly, we identified myocardium anisotropy as the driving effect on the outcomes of hyperthermic treatments. Furthermore, we characterized the complex nonlinear couplings regulating tissue behavior during RFCA, discussing model calibration, limitations, and perspectives.

## 1 Introduction

Cardiac arrhythmias are the most common and disabling pathologies worldwide (Pires et al., 1995; Kornej et al., 2020; Siontis et al., 2021) with increasing incidence–it is estimated that about 16 million people in the United States will suffer from this condition by 2050 (Patel et al., 2014). In the context of minimally invasive procedures, cardiac radiofrequency catheter ablation (RFCA) emerged as the leading clinical routine for treating cardiac arrhythmias (Nath et al., 1994). RFCA causes hyperthermic lesions destroying myocardial regions responsible for arrhythmias foci. Radiofrequency (RF) energy is carried out through a delivery antenna to target areas where the temperature is raised to at least 50°C (Wood et al., 2011). Cellular excitability is eliminated in correspondence with the ablation sites, and thermal damage is produced (Morady, 1999).

Despite the extensive clinical achievements which result in a high success rate and low mortality, RFCA is still subject to several drawbacks. An excessive thermal increase can produce steam pops and unwanted tissue lesions (Zaltieri et al., 2021). On the contrary, failure to achieve target temperatures can lead to incomplete tissue ablations, which may provoke recurrent arrhythmias that often require repeated clinical interventions. Since the temperature profoundly affects the outcome of this procedure, considerable interest has matured in predicting macroscopic thermal patterns and, in turn, estimating the expected lesion size to make RFCA risk-free and prevent failures.

Computational modeling has emerged as a feasible way to predict the outcome of cardiac RFCA procedures and to tackle specific unanswered questions. Numerical techniques, including the Finite Element Method (FEM), have been extensively used in numerous studies on cardiac RFCA (Dillon-Murphy et al., 2018; Roney et al., 2020) and, more generally, RFCA of biological tissues. Within the spectrum of RFCA, the electromagnetic source is approximated by a quasi-static form of Maxwell’s equations, often disregarding the effects of external factors. Heat transfer is usually modeled *via* the classical Pennes’ bio-heat equation due to its relative ease of implementation (Trujillo and Berjano, 2013; González-Suárez and Berjano, 2015; Park et al., 2016; Qadri et al., 2017; Petras et al., 2019). Only a few more complex models incorporating non-Fourier effects have been proposed in the literature (Tzou, 1995; Sahoo et al., 2014; Singh and Melnik, 2019). Besides, the vast majority of computational studies include simplified two-dimensional geometries (Schutt et al., 2009; Joseph and Rajappan, 2012; Hamaya et al., 2018; Singh and Melnik, 2019). The 2D nature of the current models dramatically reduces their reliability and predictability–planar domains cannot investigate cardiac microstructural features, i.e., rotational anisotropic conduction.

The role of the myocardial fibers on thermal conductive properties remains unexamined mainly, both from the experimental and computational points of view. Previous works claimed the anisotropic thermal and electrically conductive nature of the myocardium as a possible explanation for the discrepancies found between elliptical experimental/clinical lesions and the computed spherical ones (Petras et al., 2019, 2018). Only one study hitherto was found, which integrates anisotropic conductive properties in the analysis of cardiac ablation (Xie and Zemlin, 2016). Nevertheless, the work focused on pulsed-field ablation with penetrating needles and neglected tissue heat transfer. In a recent contribution (Molinari et al., 2022), we derived a novel thermo-mechanical framework for cardiac RFCA based on energetic reasoning and variational procedures also accounting for tissue microstructure. We showed that complex patterns of tissue damage and residual strains appear depending on the applied contact force and local material properties. Here, we aim at generalizing the classical thermo-electric model from the literature, considering, for the first time, a three-dimensional model of RFCA including anisotropic thermo-electrical conduction, a three-state cellular death model, and a higher-order formulation for heat transfer. In addition, we work out an experimental-based model tuning considering an innovative high-resolution measure of the thermal profile during the RFCA procedure employing Fiber Bragg Grating (FBG) sensors.

The final aim of the work is to assess the influence of the underlying myocardial microstructure on the thermal behavior at the tissue level. Accordingly, we present a multiphysics material model, implemented in a multiscale computational framework fine-tuned via multi-point temperature measurements on *ex-vivo* swine cardiac tissue undergoing RFCA. We exploited FGB sensors due to their excellent metrological and physical characteristics (i.e., small size, high thermal sensitivity, good accuracy and spatial resolution, short response time, immune to electromagnetic fields (Schena et al., 2016)), obtaining high spatio-temporal resolution of temperature maps in tissue depth. Ultimately, we integrated the space and time evolution of thermal ablation into a simplified three-dimensional finite element model of the cardiac tissue. We further considered multiscale boundary conditions, incorporating the effect of blood flow, electrical impedance, and power dissipation circuit, thus addressing and discussing several open questions of clinical relevance.

The manuscript is organized as follows. In Section 2 we provide the experimental setup developed and the RFCA protocol adopted. Besides, a description of FBGs for temperature measurements is described in terms of working principle, sensors positioning, and tissue damage estimation. In Section 3 we detail the computational model developed in terms of geometry, microstructured, and boundary conditions. We work out our generalized theory of heat transfer, further introducing an additional coupled model of thermal damage by means of cellular death and thermal dependency of material properties. Section 4 presents experimental and modeling results based on numerical convergence, sensitivity analysis, and fine-tuning of the anisotropic constitutive parameters. In particular, we discuss model accuracy and predictability quantifying the relative error between simulations and experiments. Section 5 closes the manuscript with a critical discussion of the results, highlighting limitations and perspectives.

## 2 RFCA on *Ex-Vivo* Swine Myocardium

This section describes the experimental setup and the RFCA protocol used to investigate the temperature variation (Δ*T*) in the porcine myocardium. A focus on the FBG sensors’ working principle is provided.

### 2.1 Experimental Setup and RFCA Protocol

**FIGURE 1:**
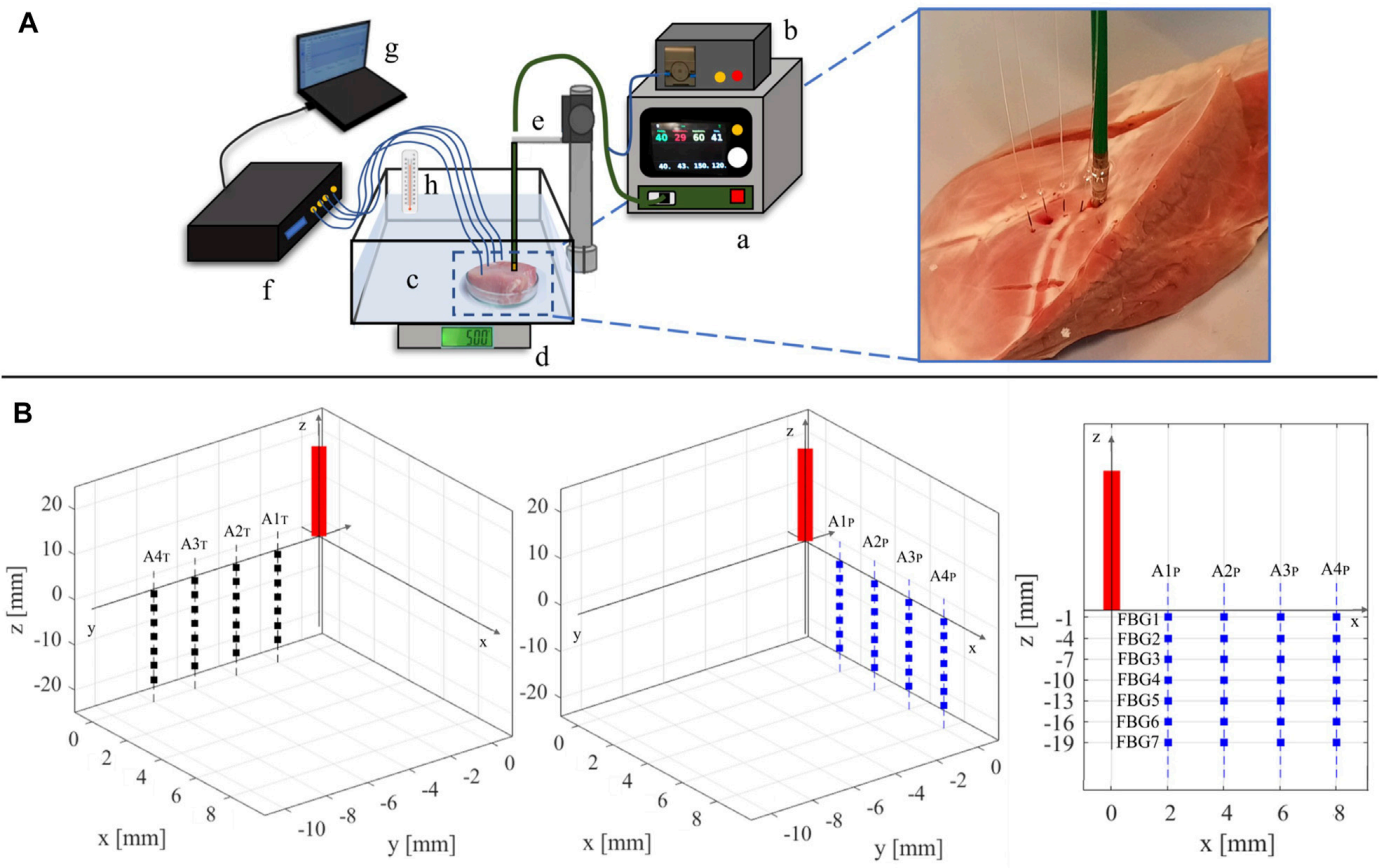
**(A)** Representation of the experimental setup: a) RF generator; b) cooling system; c) container with saline solution holding the myocardial specimen; d) digital scale; e) plexiglass RF antenna holder; f) spectrum analyzer connected to the four optical fibers; g) personal computer; h) thermometer for temperature reference. On the right, a zoom-in on the tissue specimen displaying the placement of the RF antenna and the four fiber optics is reported. **(B)** Schematic of the RF antenna and FBGs placement within the specimen. The FBGs positioning into the y-z plane (left image) and in the x-z plane (central image) with respect to the RF antenna (red rectangle) is reported. On the right, the arrangement of the FBGs in one of the two planes (x–z) is depicted as an example. The RF antenna tip is considered as the origin of the plane.

Two freshly excised swine hearts were collected from the local slaughterhouse. Three myocardial specimens with a thickness of at least 30 mm were extracted from the left ventricular area (such a thickness ensures that the RFCA treatment is confined within the ventricular wall) and put into a water bath at ≈ 37°C to achieve body temperature. Each specimen was then positioned in a container filled with saline solution, placed upon a precision digital scale (EU C7500, Gibertini Elettronica, Novate Milanese, MI). An RF antenna (FlexAbilityTM Ablation Catheter Sensor EnabledTM, Abbott Medical, MN, United States) with 2.5 mm of tip diameter and connected to a cooling system (Cool Point™, Abbott Medical, MN, United States) delivering 17 [mL/min] of saline solution was positioned on the surface of the tissue. A slight pressure was exerted on the antenna until the value of about 12 gf (i.e., about 0.118 N) was displayed by the scale, to simulate clinical pressure conditions. A perforated plexiglass positioner was exploited to hold the RF antenna in place. Four optical FBG arrays were inserted into the specimen with the help of four steel needles 20 Gauge calipered. RF impulses were produced by a RF generator (Generatore Ampere, Abbott Medical, MN, United States). A spectrum analyzer (si255 based on Hyperion Platform, Micron Optics, Atlanta, GA, United States) connected to a personal computer was employed during all the RFCAs to collect the fibers’ outputs at 1 kHz of sampling frequency. Impedance measurement was performed during the ablation procedure. The values at the starting and ending instants of each RF delivery process of the protocol described below are provided in Table 1. In Figure 1A, the experimental setup is shown.

**TABLE 1:** Impedance values at the starting (Ii) and ending (If) instants of each of the six experimental RF delivery process.

Trial	Ii [Ω]	If [Ω]
1	58	56
2	59	57
3	54.5	52
4	57	55
5	61	58
6	51	50

Two RFCAs were performed for 60 s of treatment time at 30 W of power delivery in two different points of each specimen. On every tissue sample, fibers placement and RF deliveries were executed according to the experimental protocol detailed as follows: 1) the RF antenna was set in a specific point of the tissue surface; 2) the four optical fibers were placed in a x-z plane parallel to the longitudinal axis of the antenna; 3) a first RF delivery was performed; 4) the optical fibers were removed, and the antenna was moved about 4 cm below the previous application site; 5) the four optical fibers were inserted in a y-z plane parallel to the longitudinal axis of the antenna and mutually orthogonal to the previous x-z plane; 6) a second RFCA was performed. For the sake of clarity, an example of fiber positioning is shown in Figure 1B.

### 2.2 FBGs for Temperature Measurements

Multi-point ΔT measurements were performed into swine myocardial specimens (starting from the initial tissue temperature T_0_ ≈ 37°C) during RFCA by means of four nominally identical FBG arrays (FiSens GmbH, Braunschweig, Germany). Each array enclosed 7 FBGs (acrylate coating, reflectivity value >20% , FWHM value < 2 nm, and declared thermal sensitivity S_T_ = 0.01 nm°C) of 1 mm in length and 2 mm of edge-to-edge distance (total sensing length of 19 mm), each of whose set at a specific wavelength (i.e., Bragg wavelength, *λ*_*B*_ nm) ranging from 1,500 to 1,600 nm.

#### 2.2.1 Working Principle

Once illuminated by a broadband source, an FBG operates as a notch filter which reflects a narrow spectrum of light centered around its *λ*_*B*_. As shown in (1), *λ*_*B*_ is function of the core effective refractive index (*η*_*eff*_ [⋅]) and the grating period (Λ [nm]) (Erdogan, 1997):

$$\lambda_{\text{B}}=2\eta_{\text{eff}}\Lambda$$ (1)

Both *η*_*eff*_ and Λ depend on ΔT and strain (*ϵ*). These two parameters cause a shift in the reflected spectrum, thus of *λ*_*B*_ (Δ*λ*_*B*_). As the experiments were performed in a strain-free configuration, the Δ*λ*_*B*_ was attributable to the ΔT contribute only, as also proven in a previous work (Zaltieri et al., 2020). The relationship between Δ*λ*_*B*_ and ΔT reads:

$$\frac{\Delta \lambda_{\text{B}}}{\lambda_{\text{B}}}={\text{S}}_{\text{T}}\Delta \text{T}$$ (1a)

#### 2.2.2 Sensors Positioning Within Tissue

Before each RFCA, the optical fibers were inserted into the myocardial tissue and placed into the specimen (according to anatomical landmarks), parallel to the longitudinal axis of the RF antenna and spaced 2 mm, 4 mm, 6 mm, and 8 mm apart from it. The fiber insertion was adjusted to have all the FBGs as reported in Figure 1B. For ease of reference, the optical FBG arrays are denoted with “A” associated to a number from 1 to 4, starting from the closest to the RF antenna to the farthest one. Also, the fibers belonging to the x-z plane (which is parallel to the RF antenna) are denoted by the subscript “P” (i.e., *A*1_*P*_, *A*2_*P*_, *A*3_*P*_, and *A*4_*P*_), while the ones lying in the y-z plane (which is transversal to the x-z plane) are denoted by the subscript “T” (i.e., *A*1_*T*_, *A*2_*T*_, *A*3_*T*_, and *A*4_*T*_).

The 7 FBGs embedded in each array are labelled with a progressive number from 1 to 7 (i.e., FBG1, FBG2, FBG3, FBG4, FBG5, FBG6 and FBG7), starting from the outermost to the innermost. In each plane, a total amount of 28 sensing points distributed in a tissue area of 6 mm × 19 mm are positioned.

#### 2.2.3 Hyperthermal Tissue Damage Estimation

At the end of the experimental session, the hyperthermal damage caused to the myocardial tissue by RFCA was evaluated. For each specimen, in correspondence with every ablation site, tissue was sectioned along the insertion plane of the arrays. The dimensions of each lesion (intended as the area that visually showed a lighter coloring, as common in the literature (Calzolari et al., 2017)) were manually estimated in terms of length and depth by means of a digital caliper.

## 3 Computational Model

### 3.1 Computational Domain and Myocardial Fiber Modeling

**FIGURE 2:**
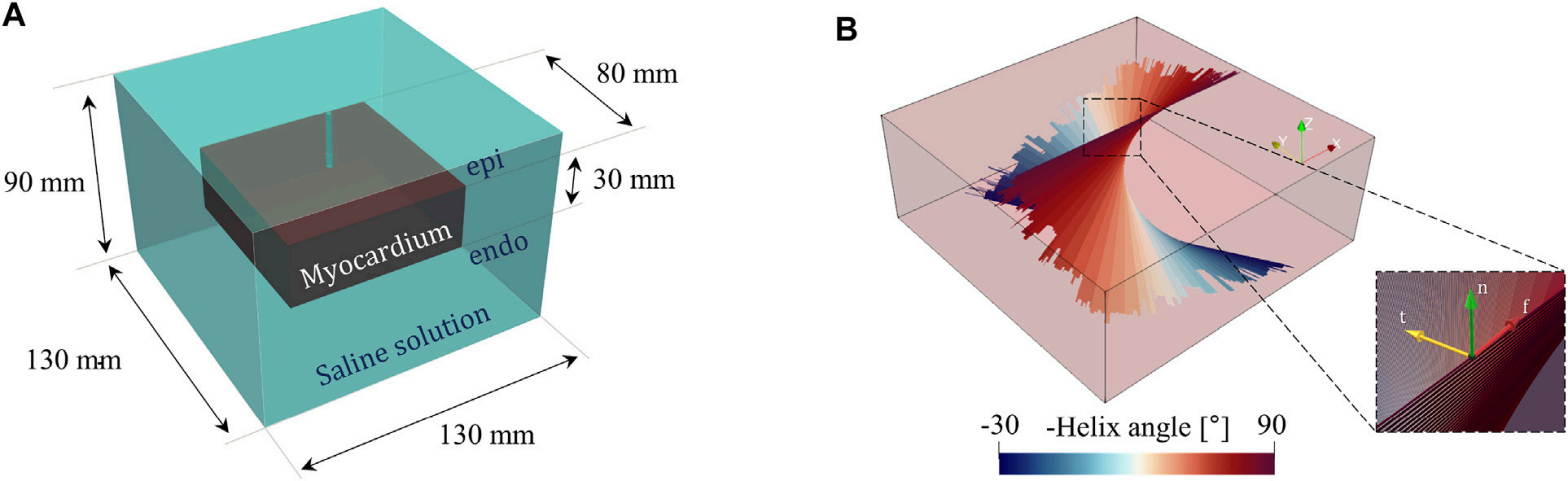
**(A)** Computational geometry, including the myocardium and saline solution domain. **(B)** 3D fiber distribution showing myocardium rotational anisotropy. The inset provides the fibers local reference system with a right-handed orthonormal set of basis vectors (**f**, **t**, **n**), where **f** denotes the muscle fiber axis, **t** the sheet (or cross–fiber) axis, and **n** the sheet–normal axis **n** = **f** ×**t**.

The computational model developed in the present work replicates the experimental setup described before by means of a simplified geometry model. We included a tissue sample of 8 cm × 8 cm × 3 cm and the surrounding domain of saline solution 13 cm × 13 cm × 9 cm (see Figure 2A). The chosen dimensions, with constant thickness, are in line with the physical dimensions of a porcine ventricular wall and do not affect the overall numerical results as discussed in the following. The catheter and electrode geometries have been omitted to reduce the overall computational burden and have been considered by appropriate thermal and electrical boundary conditions detailed in the next sections. The geometry is encapsulated in a larger box to which a coordinate scaling was applied. Therefore, these regions are treated as having an infinite extent compared to the scale length of the model, thereby minimizing boundary effects. According to well-established evidence, ventricular myocardial rotational anisotropy was implemented assuming a 120°counterclockwise rotation of the fibers, from epicardium to endocardium (Lombaert et al., 2012). To set a direct comparison with experimental data, fibers were assumed parallel to the tissue surface and aligned to the *x*-axis of the epicardial layer (see Figure 2B). We remark that myocardial fibers rotate continuously throughout the ventricular wall building up a sheet-like transverse isotropic material changing the conductivity properties along the depth. In other words, the material is both anisotropic and heterogeneous. The fiber rotational anisotropy is thus described by the law:

$$\theta \left(\text{z}\right)=\theta_{\text{epi}}+\frac{\text{z}-z_{\text{epi}}}{{\text{z}}_{\text{endo}}-{\text{z}}_{\text{pi}}}\left(\theta_{\text{endo}}-\theta_{\text{epi}}\right)$$ (2)

being *z* the thickness direction with versor **n**.

### 3.2 Multiscale Electrical Model

The electric potential due to the ablative procedure was determined by solving the time-harmonic Maxwell’s equations (quasi-static regime), complemented by the constitutive relations:

∇⋅J=0,J=σE+jωD,E=−∇V,D=εE (2a-d)

where V is the electric potential, **E** the electric field vector, **J** the current density vector, **D** the electric displacement vector, *ɛ* the electrical permittivity constant, and *ω* the angular frequency. Considering the frequency spectrum of RFCA, the wavelength of the electromagnetic field is ∼600m , several orders of magnitude larger than the spatial scale of the system, i.e., ∼10cm . Accordingly, we can assume the biological medium as resistive and solve the quasi-static form of the electrical problem.

The second-order conductivity tensor ***σ*** is introduced to model the anisotropic electrical conduction in the ventricular wall as:

$$\sigma =R\left[\begin{array}{ccc} \sigma_{\text{f}} & 0 & 0\\ 0 & \sigma_{\text{t}} & 0\\ 0 & 0 & \sigma_{\text{n}} \end{array}\right]R^{\text{T}}$$ (3)

where σ_f_, σ_t_, σ_n_ [S/m] are the conductivities in the fiber and transverse directions, respectively, and **R** = [**f t n**] is the rotation matrix tensor, based on the local fiber reference system see Figure 2B):

$$R=\left[\begin{array}{ccc} \cos\left(\theta \right) & -\sin\left(\theta \right) & 0\\ \sin\left(\theta \right) & \cos\left(\theta \right) & 0\\ 0 & 0 & 1 \end{array}\right]$$ (4)

The saline solution was modelled as an isotropic medium with constant conductivity *σ*_*sol*_.

**FIGURE 3:**
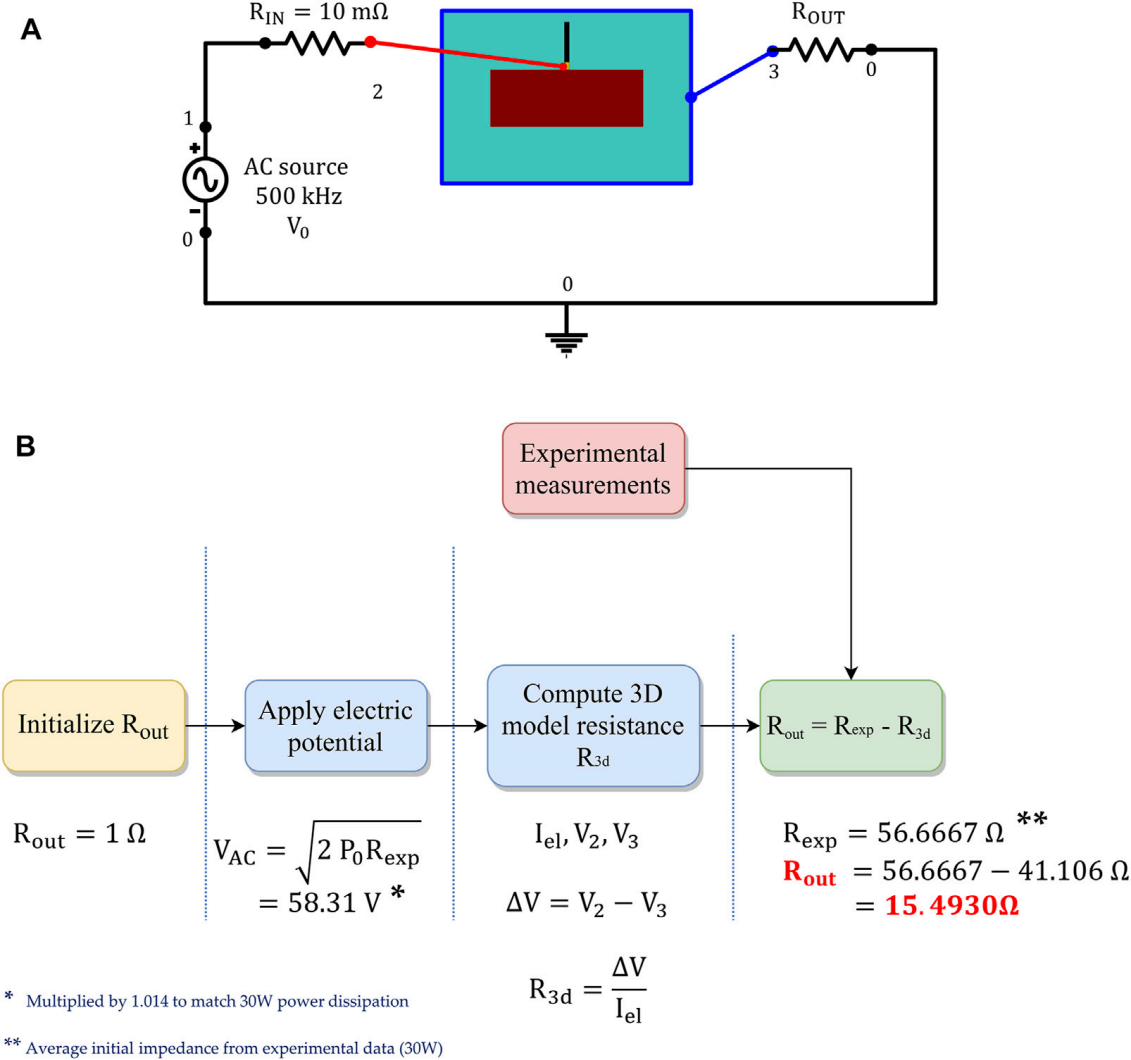
**(A)** External 0D electrical circuit coupled to the 3D computational model, to apply the RF stimulus. **(B)** Procedure employed to calibrate the output resistance *R*_*out*_, matching the experimentally measured impedance.

We further adopted a multiscale modeling approach, coupling the 3D computational domain with an external 0D electrical circuit composed of an AC voltage generator, an input, and output resistance. The latter is fine-tuned in a pre-processing step to match the experimentally measured impedance and the applied power source, according to the RFCA protocol (see Figure 3). The electric potential from the AC voltage generator follows a sigmoidal increase from 0 to $V_{0}=\sqrt{2PR_{ref}}$ in a time window of 1s, with *p* = 30 *W* being the applied power; Dirichlet boundary conditions were applied to the electrode tip $\left(V=V_{R_{in}}\right)$ and the outer surface of the saline solution $\left(V=V_{R_{out}}\right)$ to simulate the connection with the external circuit; a homogeneous Neuman boundary condition (−***σ*** ∇*V* ⋅ ***n*** = 0) was applied at the catheter surface to simulate electrical insulation.

### 3.3 Heat Transfer Model

Heat transfer was described using two alternative formulations:

$$\rho c\frac{\partial \text{T}}{\partial \text{t}}=\text{k}\nabla^{2}\text{T}$$ (5a)

$$\tau_{\text{q}}\rho c\frac{\partial^{2}\text{T}}{\partial^{2}\text{t}}+\rho c\frac{\partial \text{T}}{\partial \text{t}}=\nabla \cdot \left(k\nabla \text{T}\right)+\tau_{\text{t}}\nabla \cdot \left(k\frac{\partial \nabla \text{T}}{\partial \text{t}}\right)+{\text{Q}}_{\text{s}}$$ (5b)

where Eq. 5a refers to the parabolic heat equation based on the classical Fourier’s theory (Pennes, 1998) employed for the saline solution domain, and Eq. 5b denotes the so called dual-phase-lag (DPL) equation (Tzou, 1995), which is solved for the myocardium. Here, **T** [K] is the temperature, *ρ* [kg/m^3^] the density, *c* the heat capacity [J/kg ⋅ K], **k** [W/m ⋅ *K*] the thermal conductivity tensor, Q_s_ = **J** ⋅ **E** [W/m^3^] the external electromagnetic heating source, and *τ*_*q*_, *τ*_*t*_ [s] the phase-lag time constants.

Classical Fourier-based models fail to capture the finite thermal propagation speed due to the inherently heterogeneous microstructure of the biological medium and fast transient temperature increase occurring in hyperthermal treatments due to the small time scales and high-temperature gradients involved. Different theories have been proposed to account for non-Fourier behavior (e.g., hyperbolic heat equations and relativistic heat transfer). Among them, the so-called dual-phase-lag proposed by (Tzou, 1995) characterizes this phenomenon by means of a hyperbolic heat equation including two thermal relaxation time constants *τ*_*q*_ and *τ*_*t*_, to control the delayed response between the heat flux and temperature gradient induced in the medium. In such a framework, *τ*_*q*_ defines the time lag between the heat flux and temperature gradient, while *τ*_*t*_ governs the phase lag in establishing the temperature gradient in the conductive medium, accounting for microstructural inhomogeneities of the biological tissue. Notably, the constraint *τ*_*q*_ < *τ*_*t*_ (i.e., heat flux precedes the temperature gradient established in the tissue) must be satisfied not to violate the causality principle (Singh and Melnik, 2019). As shown in (López Molina et al., 2014), parabolic heat equation (Fourier’s theory) and hyperbolic heat conduction (DPL) converge for infinite time or infinite distance from the electrode. Our findings confirm the assumption that both heat conduction models recover the steady-state solution. In contrast, the DPL model is required to catch the initial transient temperature increase, which has a critical impact on the evolution of the damage pattern in the tissue.

**FIGURE 4:**
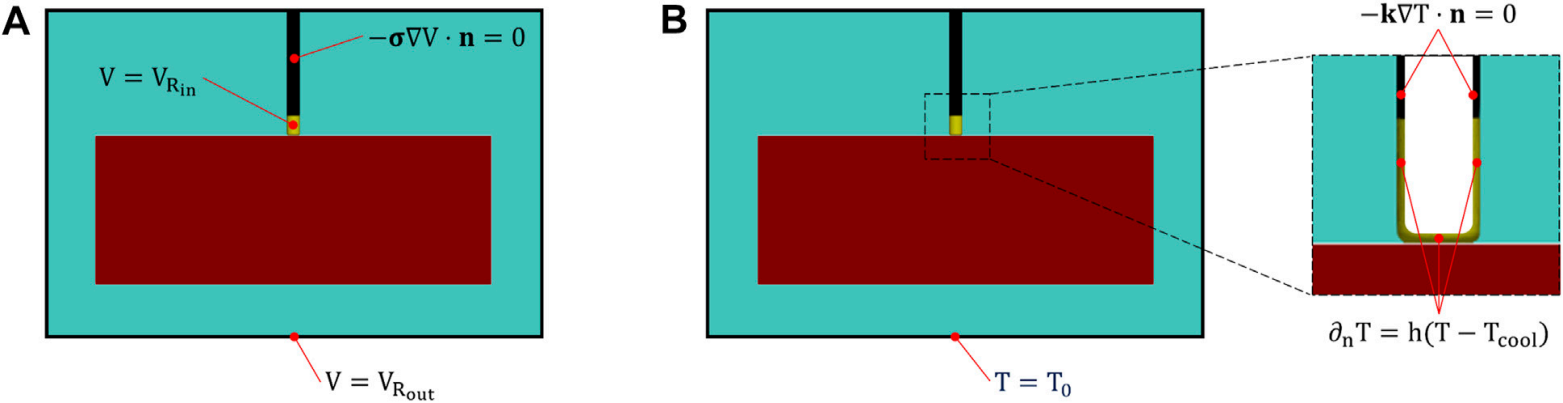
Electrical **(A)** and thermal **(B)** boundary conditions. In **(A)**, $V_{R_{in}}$ and $V_{R_{out}}$ refer to the electric potential measured at the connection node between the 3D model and the input/output resistances of the 0D circuit. In **(B)** h is the thermal convection coefficient to simulate the electrode cooling, *T*_0_ = 37 C is the reference temperature and *T*_*cool*_ = 25 C is the temperature of the coolant solution.

Consistently with the electrical problem, Eq. 3, we introduce tissue thermal anisotropy by means of the conductivity tensor ***k***, with *k*_*f*_, *k*_*t*_, *k*_*n*_, the fiber and transverse thermal conductivities, respectively; the saline solution was modeled as an isotropic medium, with a constant thermal conductivity *k*_*sol*_. According to the experimental setup, we did not consider blood perfusion in the ventricle and applied the following boundary conditions for the heat transfer model (see Figure 4): a constant temperature *T*_0_ = 37°C was imposed to the outer boundaries of the saline solution domain; thermal insulation (−**k**∇T ⋅ **n** = 0) was assumed at the catheter surface; a Robin convective boundary condition (−**k**∇T ⋅ **n** = h(T − T_c_)) was applied at the electrode-solution and electrode-tissue interface to simulate the effect of saline cooling, assuming a coolant temperature T_*c*_ = 25°C, and a heat transfer coefficient h = 5,927 [W/m^2^ ⋅ K] calculated as in (González-Suárez and Berjano, 2015) by assuming an irrigation rate of 17 [mL/min].

### 3.4 Thermal Damage Modeling

Thermal damage in ventricular tissue was assessed by using the three-state variable model of hyperthermic cell death proposed by (O’Neill et al., 2011). Such a compartment model assumes the occurrence of three cell states, i.e., alive or native (N), vulnerable or unfolded (U), and dead or denatured (D). The system allows for cell reversibility to the native state N if the thermal history is short enough (reverse transition between N and U states) and preceding irreversible damage in state D. Accordingly, the kinetic equations read:

$$\text{N}\overset{\alpha_{1}}{\underset{\alpha_{2}}{⇌}}\text{U}\overset{\alpha_{3}}{\to }\text{D},$$ (6)

resulting in a system of 3 coupled ODEs controlling the dynamics of each state, readily:

$$\frac{\text{dN}}{\text{dt}}=-\alpha_{1}\text{N}+\alpha_{2}\text{U},\;\frac{\text{dU}}{\text{dt}}=\alpha_{1}\text{N}-\left(\alpha_{2}+\alpha_{3}\right)\text{U},\;\frac{\text{dD}}{\text{dt}}=\alpha_{3}\text{U}.$$ (7)

The model must satisfy the global volumetric constraint *N* + *U* + *D* = 1 (conserved cell population). The reaction rates $\alpha_{i}=A_{i}e^{-\Delta E_{i}/\left(RT\right)}$ are temperature dependent and determined by the Arrhenius law, where Δ*E*_*i*_ [J/mol] is the activation energy, *A*_*i*_ [1/s] the frequency factor, T [K] the absolute temperature and R [J/mol ⋅ K] the universal gas constant. Consistently with previous works (Qadri et al., 2017; Singh and Melnik, 2019), such a dynamic model allows us to predict and quantify several key features of the RFCA procedure. In particular, the tissue is considered to be completely damaged if N < N_*thr*_, with N_*thr*_ a tissue-dependent threshold.

**FIGURE 5:**
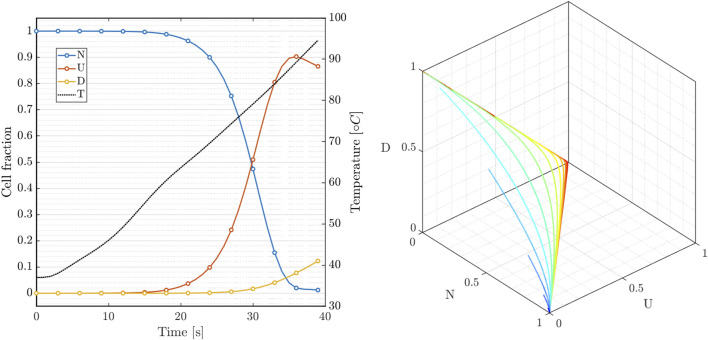
Three-state cellular model: (left) variables dynamics during RF hyperthermic treatment; (right) N-U-D phase portrait of system 7 for constant temperature applied for 800s (40 ÷ 100°C).

In Figure 5(left), we show the time course of the three state variables applying the RFCA heating curve compared with the temperature rise (black). The model replicates the nonlinear features observed experimentally and characterizes the latency of the cell death rate. Interestingly, the intermediate state balances the transition towards the irreversible dead state. Figure 5(right) also provides the phase portrait parametrized by the temperature value in the range 40 ÷ 100°*C* for 800 s. The dynamical system is characterized by the fixed point $\left[N,U,D\right]=\left[0,0,D\right]$ that changes to [0, 0, 1] once the cell population constraint is included. It is worth mentioning that the fast temperature increase due to RFCA critically modifies the reaction rates *α*_*i*_ driving the system towards the vulnerable state. Such dynamics is much faster than the system’s natural evolution towards the global fix point (dead state), enriching the nonlinear evolution of the lesion.

### 3.5 Thermal Dependency of Material Properties

Predictive simulations of RFCA have been shown to critically depend on the biophysical modeling of tissue parameters (Trujillo and Berjano, 2013). All parameters included in the simulations were gathered from literature and reported in Table 2. Consistently with the phenomenological approach adopted, we assumed temperature dependency for the following myocardial constitutive parameters (*c*, *k*, *σ*). In particular *σ* increases with temperature while *k* and *c* decrease. As a part of the model calibration, different mathematical functions were tested to describe such a temperature dependency (Petras et al., 2019):

$$\text{c}\left(\text{T}\right)=c_{0}\left[1-0.0041\left(\text{T}-{\text{T}}_{0}\right)\right]$$ (8a)

$${\text{k}}_{\text{t}}^{\text{lin}}\left(\text{T}\right)={\text{k}}_{\text{i}}^{0}\left[1-\lambda_{k}\left(\text{T}-{\text{T}}_{0}\right)\right],\text{i}=\text{f},\text{n},\text{t}$$ (8b)

$${\text{k}}_{\text{t}}^{\text{step}}\left(\text{T}\right)={\text{k}}_{\text{i}}^{0}\;\text{f}\left(\text{T}-{\text{T}}_{\text{m}},\Delta {\text{T}}_{\text{m}},\lambda_{\text{k}}\right),\text{i}=\text{f},\text{n},\text{t}$$ (8c)

$$\sigma_{\text{t}}^{\text{lin}}\left(\text{T}\right)=\sigma_{\text{i}}^{0}\left[1-\lambda_{\sigma }\left(\text{T}-{\text{T}}_{0}\right)\right],\text{i}=\text{f},\text{n},\text{t}$$ (8d)

$$\sigma_{\text{t}}^{\text{step}}\left(\text{T}\right)=\sigma_{\text{i}}^{0}\;\text{f}\left({\text{T}}_{\text{m}}-\text{T},\Delta {\text{T}}_{\text{m}},\lambda_{\sigma }\right),\text{i}=\text{f},\text{n},\text{t}$$ (8e)

where (⋅)_0_ refers to the baseline value of the parameter at body core temperature T_0_ = 37°C, *λ*_*i*_ are rates of increase/decrease, *f*(⋅, ⋅, ⋅) is a smoothed Heaviside function with a continuous second derivative, implemented via piecewise 5th-degree polynomials; the parameters T_*m*_ = 68.5°C and ΔT_*m*_ in (Eqs 8c–8e) control the step reference temperature and the transition zone, respectively. Possible generalizations of the chosen formulation are discussed in the conclusions.

**TABLE 2:** Material properties for the 3D model including the myocardium and saline solution domains.

Parameter		Value	Ref
**Electrical model**			
Myocardium fiber electrical conductivity [*S*/*m*]	$\sigma_{f}^{0}$	0.3367	Wang et al. (2001)
Myocardium transverse electrical conductivity [*S*/*m*]	$\sigma_{t}^{0}$ , $\sigma_{n}^{0}$	0.1683	Wang et al. (2001)
Saline solution electrical conductivity [*S*/*m*]	*σ*_*sol*_	1.4	Sauerheber and Heinz, (2015)
Myocardium electrical permittivity [*F*/*m*]	*ɛ*_*myo*_	3,260	(Gabriel, 1996; Raghavan et al., 2009)
Saline solution electrical permittivity [*F*/*m*]	*ɛ*_*sol*_	80	Raghavan et al. (2009)
			Maribo-Mogensen et al. (2013)
			Nörtemann et al. (1997)
**Thermal model**			
Myocardium fiber thermal conductivity [*W*/*m* ⋅ *K*]	$k_{f}^{0}$	0.6^a^	Končan et al. (2000)
Saline solution thermal conductivity [*W*/*m* ⋅ *K*]	*k*_*sol*_	0.6	Ozbek and Phillips, (1979)
Myocardium heat capacity [*J*/*kg* ⋅ *K*]	$c_{myo}^{0}$	3,017	Petras et al. (2019)
Saline solution heat capacity [*J*/*kg* ⋅ *K*]	*c*_*sol*_	4,178	Kho et al. (2021)
Myocardium density [*kg*/*m* ^3^]	*ρ*_*myo*_	1,076	Petras et al. (2019)
Saline solution density [*kg*/*m* ^3^]	*ρ*_*sol*_	997	Kho et al. (2021)
Relaxation time heat flux [*s*]	*τ*_*q*_	8^a^	Sahoo et al. (2014)
Relaxation time temperature gradient [*s*]	*τ*_*t*_	0.045^a^	Sahoo et al. (2014)
**Cell-death model**			
Frequency factor *N* → *U* [1/*s*]	*A*_1_	3.68 × 10^30^	Park et al. (2016)
Frequency factor *U* → *D* [1/*s*]	*A*_2_	5.68 × 10^3^	Park et al. (2016)
Frequency factor *U* → *N* [1/*s*]	*A*_3_	2.58 × 10^5^	Park et al. (2016)
Activation energy *N* → *U* [*J*/*mol*]	Δ*E*_1_	210, ×, 10^3^	Park et al. (2016)
Activation energy *U* → *D* [*J*/*mol*]	Δ*E*_2_	38.6 × 10^3^	Park et al. (2016)
Activation energy *U* → *N* [*J*/*mol*]	Δ*E*_3_	47.2 × 10^3^	Park et al. (2016)

a Parameter included in the parametric analysis.

## 4 Results and Discussion

### 4.1 Experimental Results

**FIGURE 6:**
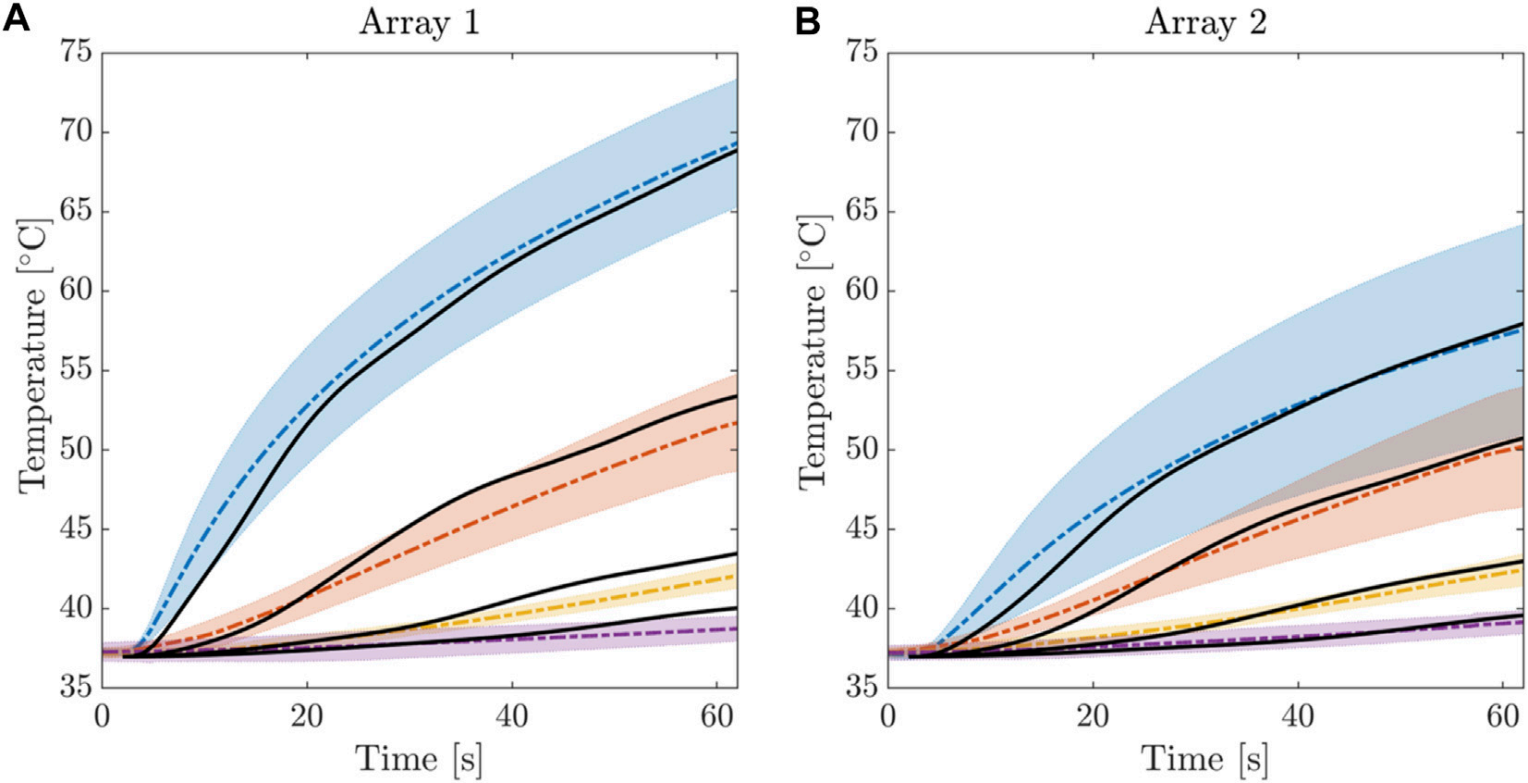
Electrical Temperature evolution during the RFCA treatment for the first 4 FBGs of Array 1 **(A)** and Array 2 **(B)**. Average temperature and confidence bands were computed from the 6 experimental trials. Solid black line indicates the results of the calibrated computational model.

FBGs data were exported and processed in MATLAB^®^ (Mathworks, Natick, MA, United States) environment. For each experiment, the ΔT trends estimated by the 28 FBGs embedded in the four arrays are analyzed with respect to T_0_ during the 60 s of RFCA. In the Supplementary Material (SM), the plots showing the temperature increase caused by the RF heating effect are reported for all the six experiments. The ΔT measured by each FBG strictly depends on its placement with respect to the RF antenna tip. In fact, FBGs positioned closer to the antenna (see Figure 1B, right image), measured higher ΔT than the distant ones, also exhibiting faster temperature rises. For example, among the sensors belonging to the same array (e.g., from FBG1 to FBG7 of A1_*T*_), FBG1 always measures the maximal ΔT values. Also, proceeding deeper into the tissue from FBG1 to FBG7, the obtained ΔT trends decrease. Furthermore, referring to FBGs placed into the tissue at the same depth but belonging to different arrays (e.g., all the FBG1s of A1_*T*_, A2_*T*_, A3_*T*_, and A4_*T*_), the ΔT trends are progressively lower starting from A1_*T*_ to A4_*T*_. For the sake of completeness, for each FBG the average temperature was computed across the six experiments, together with the standard deviation. Figure 6 shows the average temperature trends relative to FBG1, FBG2, FBG3 and FBG4 of the arrays A1 and A2, respectively. Such multi-point measurement enabled the identification and quantification of two-dimensional temperature maps to depict the temperature spatial distribution into the treated tissues at the end of each RFCA. More precisely, FBGs data were collected from the start of treatment to the moment of RF discharge. A total amount of 28 experimental values related to the 28 measurement sites as previously depicted were obtained. A linear interpolation was implemented along such temperature values and 2D experimental temperature maps were produced for all the performed RFCAs, both along x-z and y-z planes.

We remark that statistical averages were derived from the whole data set to represent a robust information bandwidth to fit the computational model. As described in the following, the tuning procedure was developed such to identify the optimal anisotropy ratio matching average and variance in tissue depth at different locations.

**FIGURE 7:**
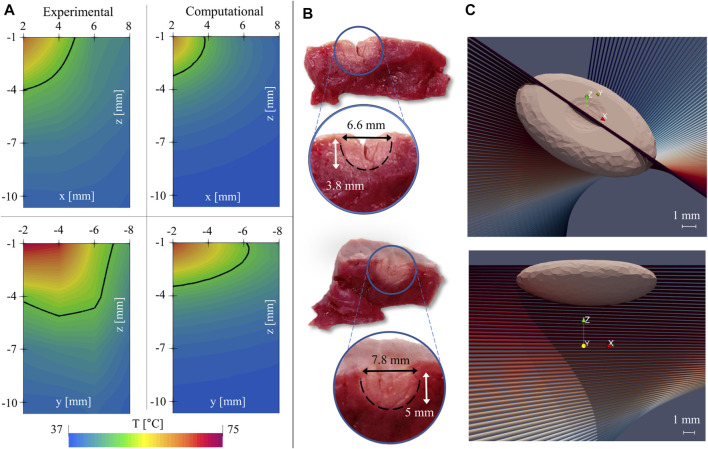
**(A)** comparison of experimental (left) and computational (right) temperature maps, revealing different elongations of the thermal lesion across orthogonal directions. **(B)** Half-sections of the tissue after RFA treatment, sliced at the lesion site, along the plane of the FBGs’ arrays. **(C)** 3D views of the numerical lesion resulting from the calibrated model.

In Figure 7A, two representative experimental temperature maps showing the temperature distributions at the final instant of the treatment (i.e., 60th s) are provided.

The temperature maps evaluated on the x-z plane (top) and on the y-z plane (bottom), and the isothermal level at T = 50°C (solid black curve) are reported. Temperatures greater than 50°C are considered responsible of myocardial tissue permanent injury. As consequence, it is supposed that the area included into the curve may be representative of the damaged tissue (Zhang et al., 2016). However, the two temperature maps differ in terms of dimension and size of the tissue area surrounded by the 50°C isotherm (which is larger in the y-z plane with respect to the x-z plane). These results confirm the occurrence of a preferential direction along which the heat diffuses. In this specific case, heat transport is higher along the *y*-axis (Figure 7A, bottom) than the *x*-axis (Figure 7A, top). Moreover, heat spreads mostly in the y-z plane than in the x-z plane. We remark that the observed anisotropic evolutions critically depend on FGB location with respect to ventricular microstructure. However, though following anatomical landmarks, FGB positioning does not get information on the underlying fiber structure. Moreover, the in-depth measure along the seven sensors gathers thermal information from twisted myocardial fibers (see Figure 1). The definition of the appropriate myocardial anisotropy requires then an accurate parametric tuning as described in the following section.

Such findings are supported by comparing the hyperthermal damages’ dimensions obtained manually by means of the digital caliper. In Figure 7B, the two images of the produced lesions sectioned along the x-z plane (upper image) and y-z plane (down image) are shown. As expected, both length and depth of the damage evaluated in the y-z plane are greater than the ones evaluated in the x-z plane (i.e., 7.8 mm vs. 6.6. mm in length, 5 vs. 3.8 mm in depth). In both cases, the lesions which were manually assessed result in larger dimensions than the ones obtained from the 2D temperature maps by using the thermal isocontour method: for instance, widths were 6.6 and 7.8 mm (Figure 7B) vs. almost 10 and 18 mm (Figure 7A), respectively. Two main motivations can justify such a discrepancy: 1) the occurrence of measurement errors caused by possible inaccuracies in the visual evaluation of the damaged area as there is no unequivocal method for visually assessing the extent of the lesions (no evidence is given that the damaged area uniquely consists of the zone with lighter color), and 2) the adoption of the isothermal curve at 50°C as the sole criterion for the lesion evaluation. Significantly, using this approach, the lesion is determined only based on the local temperature and does not account for the effects of the thermal treatment duration. In addition, there have been concerns about the actual value of the temperature threshold, and various alternatives have been proposed in the literature, e.g., 55 and 59°C (Zhang et al., 2016). To cope with such a non-unique definition, in the following, we provide a rationale based on the three-state dynamics cell model.

### 4.2 Model Setup and Convergence Analysis

Numerical simulations were performed with the finite element software COMSOL Multiphysics^®^ (Comsol 5.6, COMSOL, Stockholm, Sweden). We discretized the computational domain with mixed linear tetrahedral and hexahedral elements. In particular, hexahedral elements were used to discretize the additional domain for the coordinate scaling (see Section 2.1), with a significantly reduced number of elements in the mesh. From a relatively coarse discretization, the mesh was gradually refined near the electrode area, which is strongly affected by hyperthermic treatment. To solve the problem in the time domain, an implicit BDF scheme with adaptive time stepping was adopted. A preliminary convergence analysis ensured that the results were independent of the numerical approximation. Specifically, five different meshes were tested, and the optimal discretization (71,501 degrees of freedom) was chosen to achieve an error below 1*%* for the maximum temperature of each FBG, compared to the reference solution, i.e., the one with the finest mesh (see Molinari et al. (2021)).

### 4.3 Sensitivity Analysis and Model Calibration

In the following, we present the extensive sensitivity analysis performed to fine-tune the computational model and match experimental data. Virtual probes were positioned in the computational model following FBG positions in the experimental setup to obtain consistency for quantitative comparison. Initially, a preliminary model tuning was conducted using the parabolic Fourier model Eq. 5a getting a basal thermo-electric profile in the myocardium. Then, the DPL model Eq. 5b was introduced to better replicate the transient temperature rise during RFCA treatment. Finally, the parameters for anisotropic thermal conductivity were adjusted to match the spatial temperature distribution obtained from the multi-point measurements. For the sake of conciseness, the complete set of numerical analyses are available at (Molinari et al., 2021). In the following, we will rather present how the calibration process was carried out and discuss the main findings of the study.

#### 4.3.1 Electrical Model Tuning

Different functional forms for the electrical conductivity were tested, as described in Section 3.5, with the linear function resulting in better compatibility with reference data. The linear increase rate *λ*_*σ*_ was calibrated to achieve an impedance drop at the end of ablation in the range of experimental measurements (2 ± 0.63 Ω). A value of *λ*_*σ*_ = 0.1 was selected and included in the upcoming analyses.

#### 4.3.2 Fourier Thermal Model Tuning

A preliminary numerical examination confirmed that the baseline value of thermal conductivity k_0_ = 0.6 [W/m ⋅ K], gathered from literature, provided optimal results in terms of temperature distribution within the tissue. Afterwards, we calibrated the two proposed functions for thermal conductivity, i.e., smoothed step (ΔT_*m*_ = 55°C, *λ*_*k*_ = −0.006) and linear (*λ*_*k*_ = −0.001). Results comparison shows that both strategies furnish similar values, albeit with significantly reduced computational time in the case of the linear decrease of *k*. Accordingly, we assumed a linear temperature dependence for the thermal conductivity.

#### 4.3.3 DPL Model Time Constant Tuning

After calibrating the myocardium’s thermal and electrical constitutive properties using the parabolic heat conduction, we investigated the effect of phase lag on the model outcome. To handle separately the two phase-lagging phenomena reproduced in the model (i.e., heat flux and temperature gradient delays), we performed two different tests by modifying the value of one of the time constants while keeping the other fixed. Our investigations determined that the values reported in (Sahoo et al., 2014) (*τ*_*q*_ = 8 s, *τ*_*t*_ = 0.045 s) best fit our experimental data.

#### 4.3.4 Thermal Anisotropy Tuning

**FIGURE 8:**
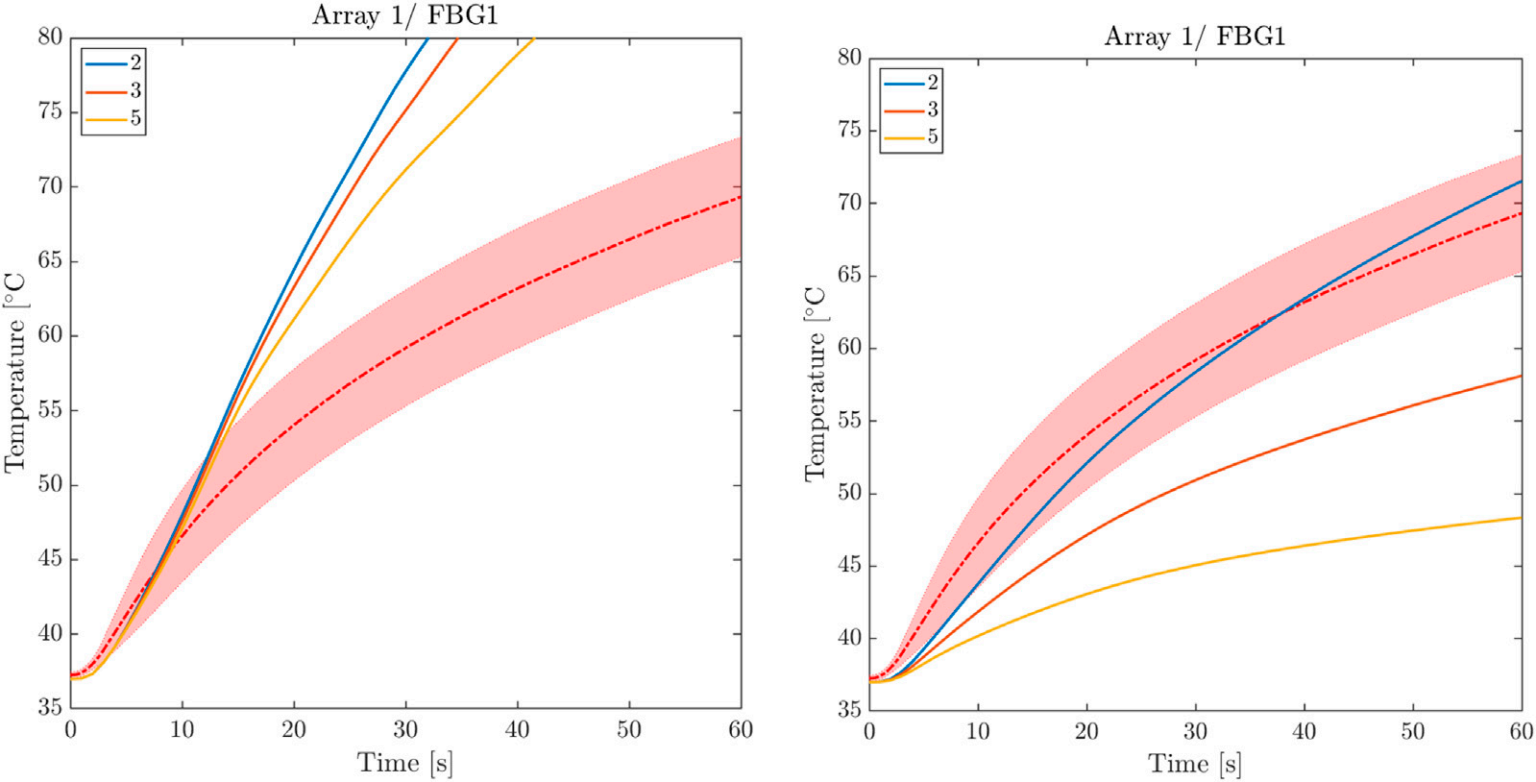
Comparison between thermal and electrical anisotropy fitting. Left: thermal isotropy for three levels of electrical anisotropy. Right: electrical isotropy for three levels of thermal anisotropy.

Experimental results outlined in Section 4.1 showed a higher temperature increase in the longitudinal direction than in the tissue depth due to myocardial fiber architecture. To achieve optimal agreement with the time course of temperature curves, we performed multiple numerical tests by varying the coefficients k_f_ and k_n_ introduced in Section 3.3, i.e., the heat conduction values in the fiber and sheet-normal directions, respectively. Starting from an isotropic conductive medium (i.e., k_f_ = k_t_ = k_n_ = k), multiple combinations were tested, with increased conductivity along the fibers (k_f_ = 2k, 3k, 5k, 6k) and decreased conductivity in the sheet-normal direction (k_n_ = 0.8k, 0.7k, 0.6k, 0.5k), holding **k** = **k**(T) the thermal dependent conductivity tensor as in Eq. 8b. Our parametric analysis revealed negligible sensitivity to the cross-fiber conductivity *k*_*t*_, and we identified [k_f_, k_t_, k_n_] = [6k, k, 0.5k] as the optimal values that provide the best fit for the experimental temperature curves. We note that, during the optimization process, thermal anisotropy mainly influences the initial temperature increase. In contrast, both contributions, electrical and thermal anisotropies, are required to reproduce experimental transient temperature increases and steady-state values. As a representative example, Figure 8 compares thermal and electrical anisotropy fitting for different ratios versus the corresponding isotropic case.

A conclusive study was conducted to test the sensitivity of the computational model to the alignment of the FBG arrays and myocardial fibers. We tested five different fiber distributions by gradually rotating the fibers at epicardial level by 10°. As expected, due to the higher conductivity in the fiber direction, we noticed a decrease in temperature as the level of misalignment increases. However, this effect was negligible, particularly for FBGs that are distant from the RF antenna. We remark that FBG arrays have been placed according to anatomical landmarks on the surface of the ventricular surface and the adopted fitting procedure considered the statistical average-variance over the total experiments. Accordingly, the conducted parametric analysis was able to identify the rotational anisotropy ratio recovering the temperature rise with negligible error (see next Section).

### 4.4 Model Accuracy and Damage Prediction

**FIGURE 9:**
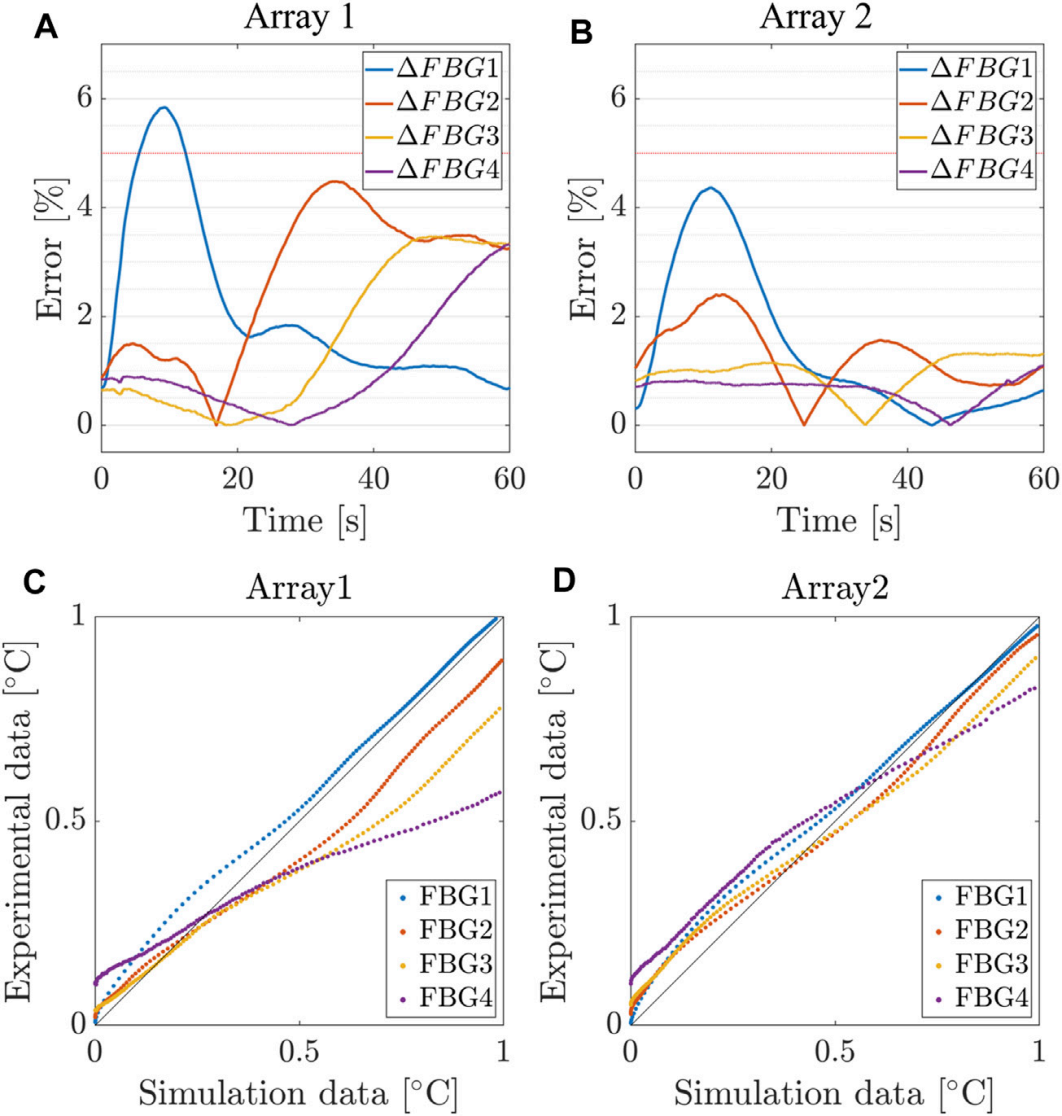
Relative error between simulated and measured temperatures for the first 4 FBGs of Array 1, 2. **(A,B)** Relative error and **(C,D)** Pearson correlation coefficient between the numerical and the average experimental temperature at each instant of the RFCA.

We present the results obtained from the calibrated model, which are thoroughly compared with the experimental counterpart. In Figure 6, we provide the time evolution of the simulated temperature profiles (solid black) over imposed to the average value and confidence bands of the experimental recordings (dashed color) for each FBG. Though clear accordance among calibrated model and *ex-vivo* measurements is obtained, we quantified the goodness of fit using two different strategies. First, we computed the relative error (Δ*FBG*_*i*_) between the numerical and the average experimental temperature at each instant of the RFCA (Figures 9A, B). Additionally, we analyzed the relationship between experimental and simulated temperatures using the Pearson correlation coefficient *ρ* (Figure 9 C, D). The plots display the statistical analysis results for the first FBGs of A1 and A2. The error settles below 5*%* for each FBG of the two arrays, with a slightly higher value (6*%*) for FBG1/A1 in the initial phase of the RFCA treatment. However, given the non-critical temperature (below 43°C) experienced by the tissue, we reasonably considered this result of minor importance.

As confirmed in Table 3, we found an excellent correlation between the predicted and experimental data. More significant relative errors, still below 10*%*, were found for FBG1 and FBG2 of the remaining arrays, while the correlation remained positive ( $\hat{\rho }\;>$ 0.98). Nevertheless, given the distance of the FBGs from the ablation site, the absolute temperature increase was limited (below 43°C). Therefore the marginal discrepancies between the model and the measurements were not considered meaningful. The complete results, including the 28 FBGs traces, are provided in (Molinari et al., 2021).

**TABLE 3:** Maximum absolute error and correlation coefficients for the simulated and measured temperatures for the first 4 FBGs of Array 1 and 2.

FBG	Max |Δ*FBG*| [*%*]	$\hat{\rho }$
Array 1		
FBG1	5.84	0.9987
FBG2	4.48	0.9972
FBG3	3.47	0.9963
FBG4	3.32	0.9934
Array 1		
FBG1	4.36	0.9979
FBG2	2.40	0.9976
FBG3	1.32	0.9978
FBG4	1.10	0.9915

**FIGURE 10:**
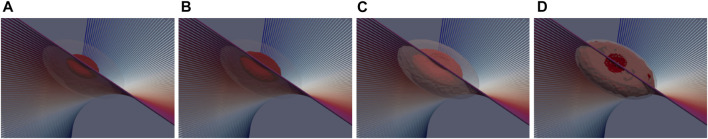
3D views of the numerical lesion resulting from the three-state cell model for different threshold levels N_*thr*_: **(A)** 0.8, **(B)** 0.9, **(C)** 0.95, and **(D)** 0.99. A shaded view of the 50°C thermal isocontour method is provided for comparison.

Finally, we provide a quantitative analysis of damage assessment and lesion prediction criterium. The lesion was first computed with the 50°C thermal isocontour method (consistently with the visual post-processing of experimental data). The computed damage volume, shown in Figure 7, is 12.6 mm in width and 3.6 mm in depth. Both experimental and computational lesions exhibit an ellipsoidal shape, with the major axis oriented towards the muscular fibers. Then, a numerical sensitivity analysis was performed by using the cell-death model varying the threshold value N_*thr*_ ∈ [0.8 ÷ 0.99]. Figure 10 shows how the cell-based computed damage volume grows as the threshold increases also depending on the ablation time. We identify N_*thr*_ ≈ 0.95 ÷ 0.98 as the optimal value for the present model vs. experimental lesions. We further note that the computational lesion follows the fiber rotation across the tissue and that the irreversible damage volume concentrates below the tissue surface according to the expected RF concentration in the presence of blood flow and saline boundary conditions. This result broadly supports the work of (Xie and Zemlin, 2016) though an extended validation in this direction is required.

## 5 Discussion

In the present study, we developed an experimentally calibrated finite element computational model of *ex-vivo* cardiac RFCA. The key innovative features of the proposed methodology are the inclusion of three-dimensional myocardial anisotropy, the implementation of a multi-scale time lag thermal ablation model, and the adoption of a dynamical model of cell death for the estimation of tissue damage and lesion sizing.

Remarkably, model calibration relies on accurate multi-point temperature measurements obtained via FBG sensors. The study offered novel insights into the anisotropic thermal behavior of cardiac tissue, which was poorly addressed in the literature. In a combined experimental-computational modeling framework, we proved that the intrinsic anisotropic microstructure of the myocardium plays a pivotal role in the thermo-electrical response of the tissue undergoing hyperthermic treatments. Furthermore, we showed that a comprehensive sensitivity analysis must comply with complex nonlinear couplings and heretogeneous materials properties.

### 5.1 Limitations

Limitations to this study need to be acknowledged. First, the computational domain was idealized. Experimental thickness variation, and associated microstructure, may represent a possible source of error in model tuning. However, a priori sensitivity analysis of varying tissue thickness indicated that overall results do not change since temperature decreases rapidly with tissue depth and significant heating only occurs in the vicinity of the electrode. Because of clinical applications, however, a patient-specific image-based realistic geometry will be mandatory for maximizing model reliability and further improving its predictability (Sung et al., 2021).

Second, material modeling lacks of cardiac electro-mechanical couplings (Singh and Melnik, 2019; Pandolfi et al., 2016, 2017; Gizzi et al., 2015; Ruiz-Baier et al., 2020), as well as tissue-electrode contact mechanics (Petras et al., 2018). Besides, three-dimensional blood hemodynamics and saline irrigation (accounted in the present study by means of multiscale boundary conditions) shall be considered in line with recent computational studies (Petras et al., 2019). We remark that our experimental calibration is performed on *ex-vivo* samples, with no external perfusion. Accordingly, we did not include the perfusion term in the heat equation to be consistent with our experimental dataset. However, additional volumetric sources (accounting for blood perfusion, metabolism, to name a few) and multi-field coupling phenomena should be included when modeling *in-vivo* cardiac ablation. In addition state-of-the-art machine learning algorithms and data assimilation procedures (Barone et al., 2020a,b; Zaman et al., 2021) are also foreseen in view of a patient-specific optimization applications (Lopez-Perez et al., 2019; Aronis et al., 2021).

From the experimental point of view, it was not possible to ensure an identical placement of the sensors within the tissues for all the experiments and no measurements were performed contemporary in the x-y plane due to difficulties in sensors positioning. We opted for a solution minimizing the positioning error of the FBGs caused by the manual insertion of the optical fibers. A dedicated 3D-printed holding setup for FBG insertion is a route we are exploring for further improving the current measurement technique.

Finally, more objective criteria for the estimation of tissue damage taking advantage of histological techniques are expected. We investigated a smooth transition of the damage criterium through a parametric analysis of the threshold value. In view of generalized robust and reliable multiscale modeling approaches, an extended validation of the damage criterium is foreseen to cope with a broad spectrum of clinical applications.

## 5.2 Conclusion

The present work has the ultimate purpose of improving the predictive capabilities of RFCA computational models in view of personalized therapy planning, design, and development of new RFCA systems and protocols and ultimately paving the way to a dramatic improvement in the safety of clinical procedures.

## Data Availability

The original contributions presented in the study are included in the article/Supplementary Material, further inquiries can be directed to the corresponding author.
