# Supplementary material for: Multiscale and Multiphysics Modeling of Anisotropic Cardiac RFCA: Experimental-Based Model Calibration via Multi-Point Temperature Measurements
Source: Front Physiol. 2022 Apr 19;13:845896. doi: 10.3389/fphys.2022.845896 (PMC9062295; doi:10.3389/fphys.2022.845896)
Supplement: Supplementary file 1 [file DataSheet2.PDF]

Multiscale and Multiphysics Modeling of Anisotropic Cardiac RFCA: Experimental-based Model Calibration via Multi-Point Temperature Measurements

Supplementary Materials

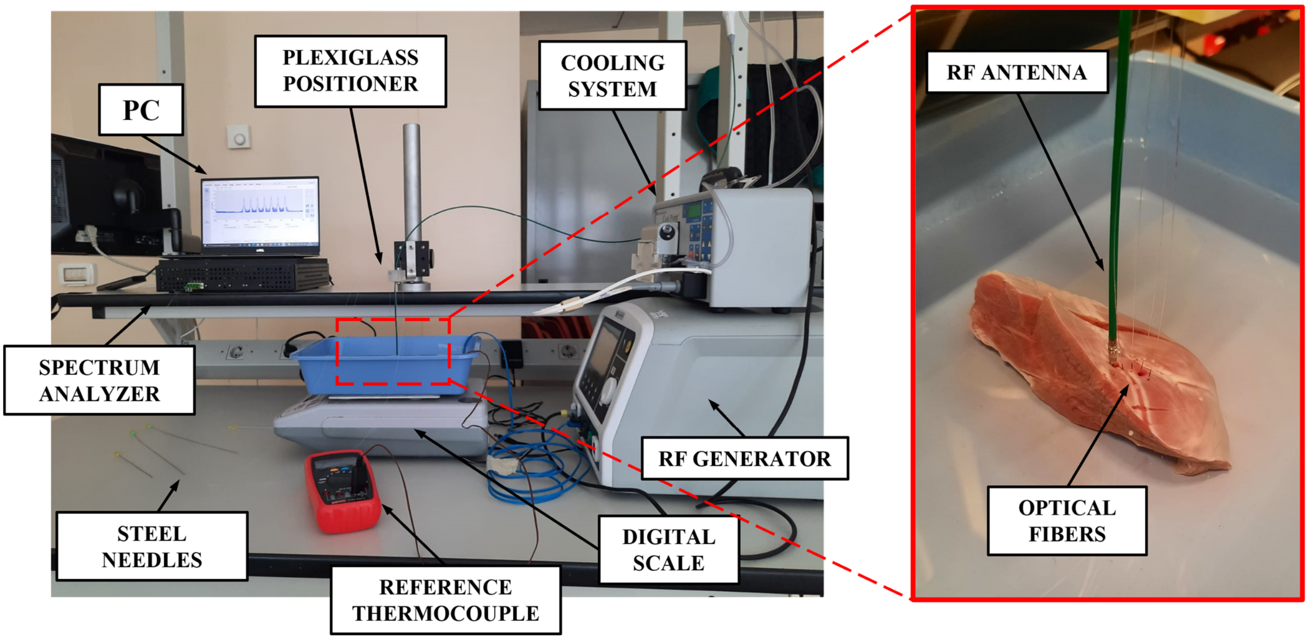

Fig. 1. SM The experimental setup. On the left, picture showing the experimental setting. On the right, a focus on the myocardial specimen. The RF antenna and the four optical fibers are shown.

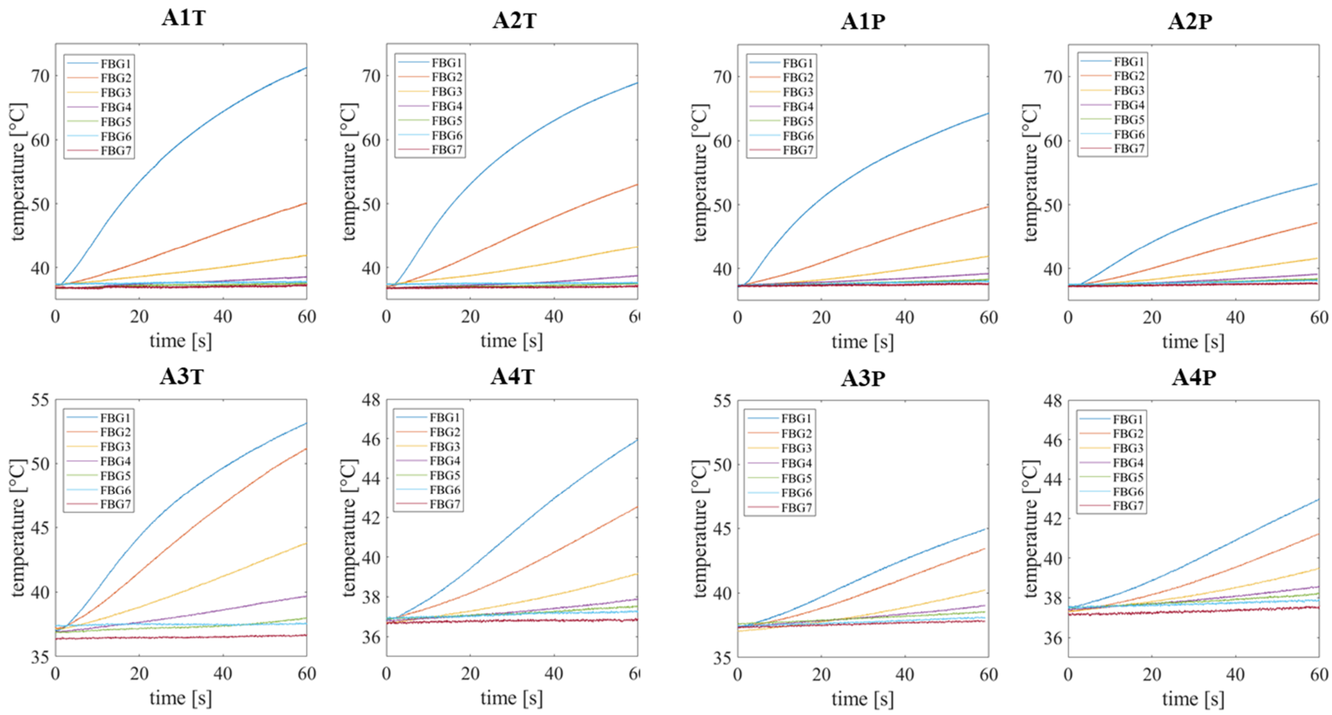

Fig. 2. SM Plots showing the temperature increases measured by the seven FBGs (FBG1, FBG2, FBG3, FBG4, FBG5, FBG6 and FBG7) belonging to the four arrays in the parallel ($A1_P$, $A2_P$, $A3_P$ and $A4_P$) and transversal ($A1_T$, $A2_T$, $A3_T$ and $A4_T$) configuration during the two RFCAs performed on the first specimen.

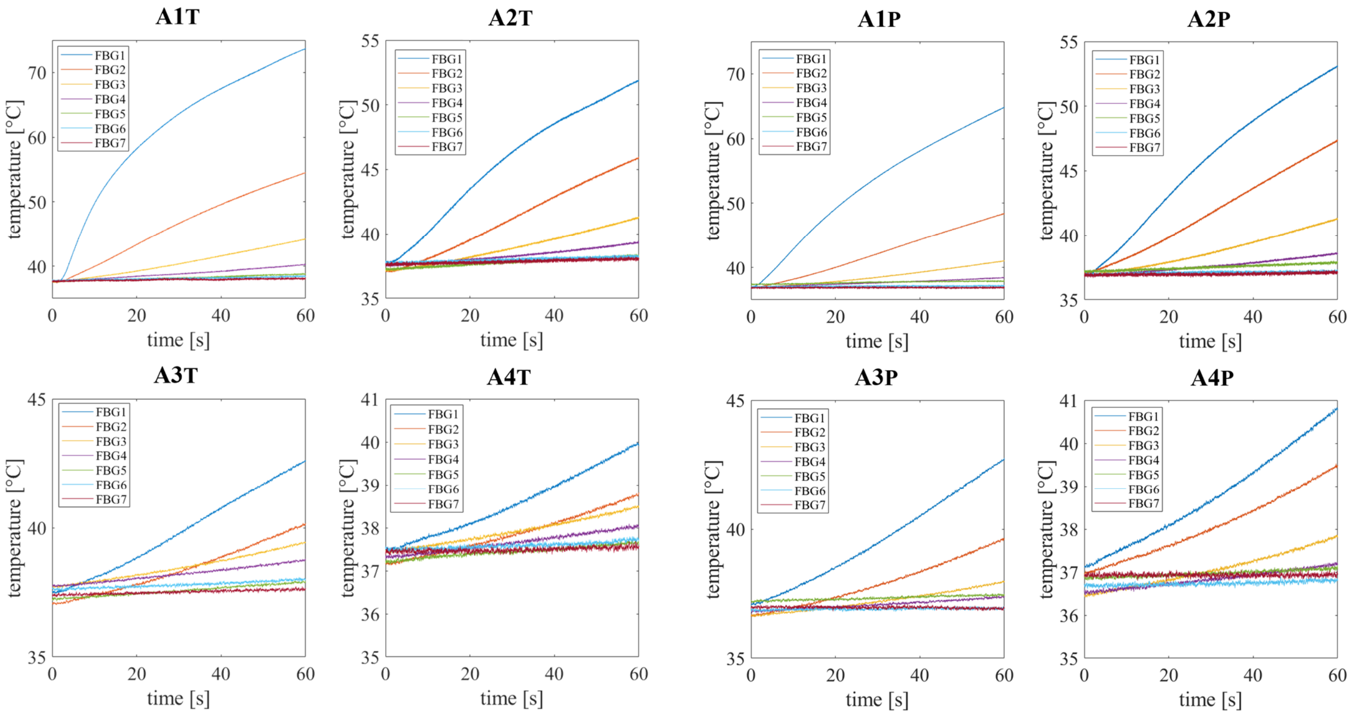

Fig. 3. SM Plots showing the temperature increases measured by the seven FBGs (FBG1, FBG2, FBG3, FBG4, FBG5, FBG6 and FBG7) belonging to the four arrays in the parallel ($A1_P$, $A2_P$, $A3_P$ and $A4_P$) and transversal ($A1_T$, $A2_T$, $A3_T$ and $A4_T$) configuration during the two RFCAs performed on the second specimen.

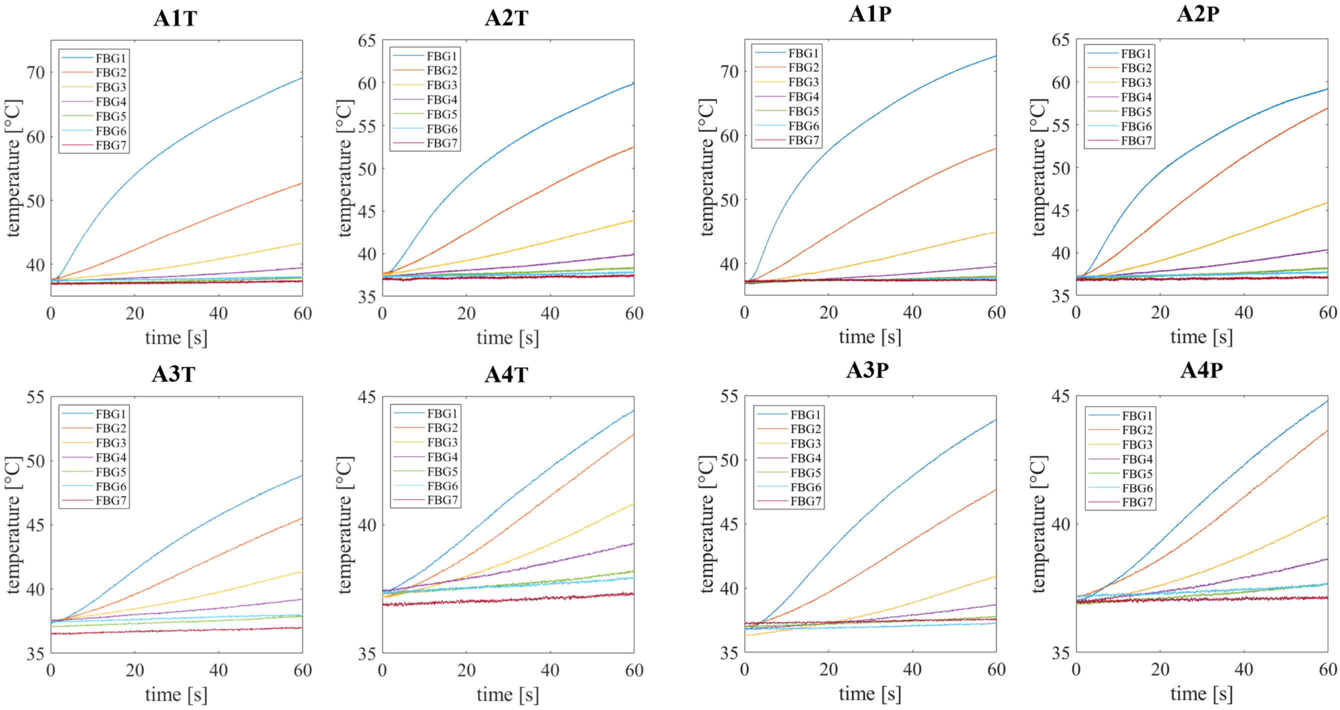

Fig. 4. SM Plots showing the temperature increases measured by the seven FBGs (FBG1, FBG2, FBG3, FBG4, FBG5, FBG6 and FBG7) belonging to the four arrays in the parallel ($A1_P$, $A2_P$, $A3_P$ and $A4_P$) and transversal ($A1_T$, $A2_T$, $A3_T$ and $A4_T$) configuration during the two RFCAs performed on the third specimen.

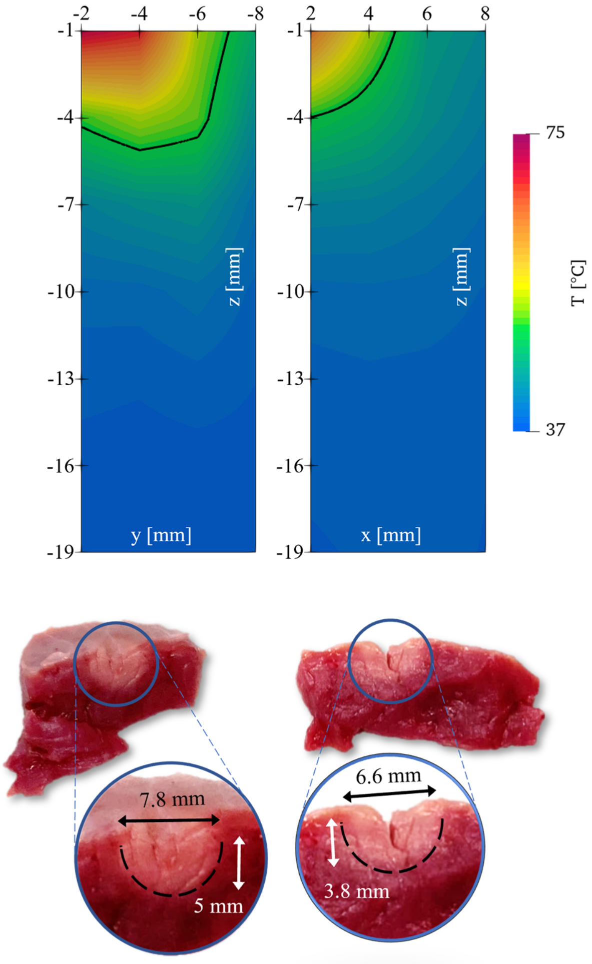

Fig. 5. SM Experimental temperature maps showing the temperature distributions along the x-z plane and y-z plane at the final instant of the treatment (i.e., 60th s). The two maps are related to the RFCAs performed on the first specimen. Below, the two lesions obtained are reported, together with their dimensions.

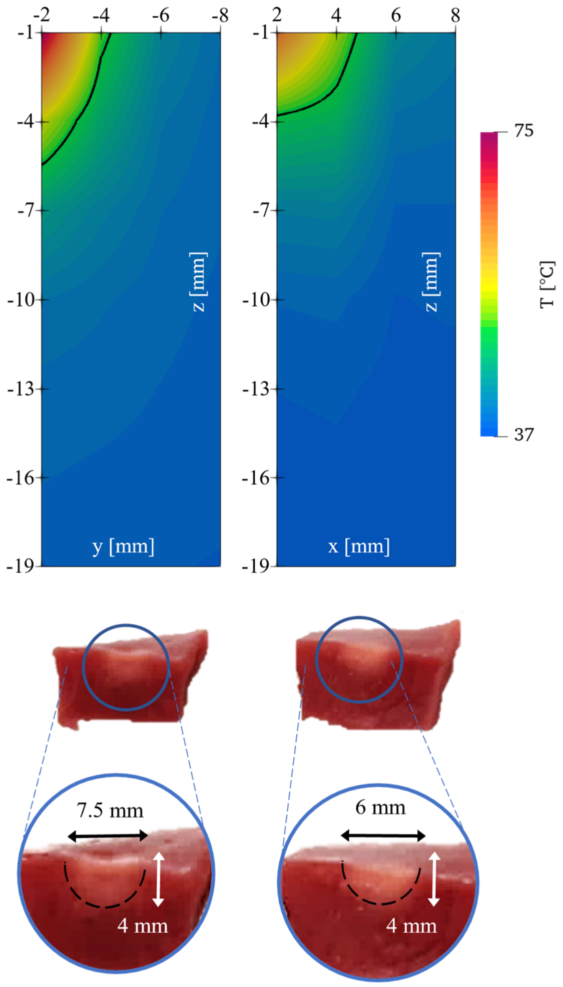

Fig. 6. SM Experimental temperature maps showing the temperature distributions along the x-z plane and y-z plane at the final instant of the treatment (i.e., 60th s). The two maps are related to the RFCAs performed on the second specimen. Below, the two lesions obtained are reported, together with their dimensions.

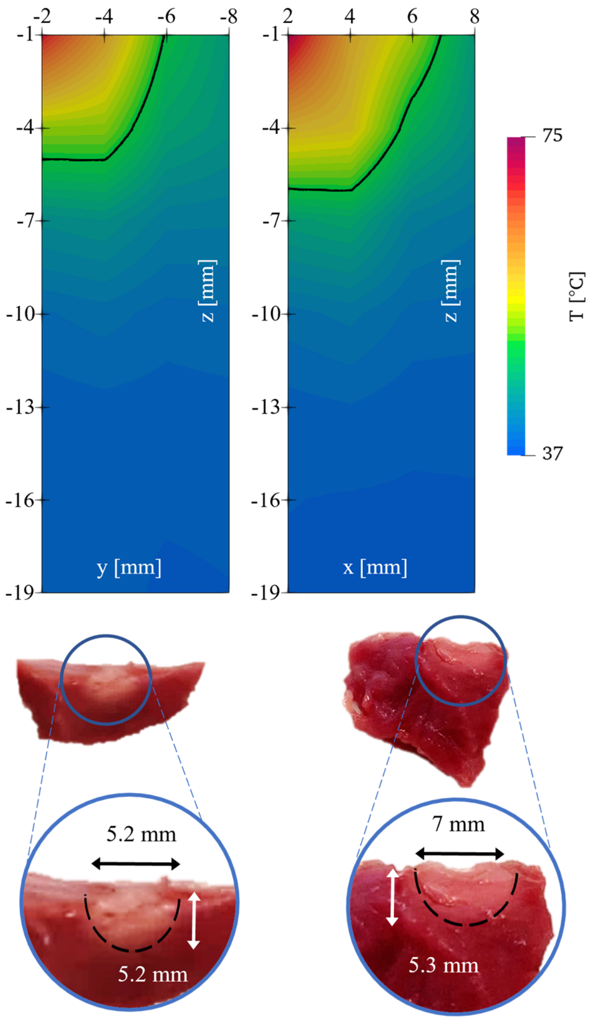

Fig. 7. SM Experimental temperature maps showing the temperature distributions along the x-z plane and y-z plane at the final instant of the treatment (i.e., 60th s). The two maps are related to the RFCAs performed on the third specimen. Below, the two lesions obtained are reported, together with their dimensions.
